# Sorption of Ochratoxin A from Aqueous Solutions Using β-Cyclodextrin-Polyurethane Polymer

**DOI:** 10.3390/toxins4020098

**Published:** 2012-02-06

**Authors:** Michael Appell, Michael A. Jackson

**Affiliations:** 1 Bacterial Foodborne Pathogens and Mycology Research Unit, United States Department of Agriculture, Agricultural Research Service, National Center for Agricultural Utilization Research, 1815 N. University St., Peoria, IL 61604, USA; 2 Renewable Product Technology Research Unit, United States Department of Agriculture, Agricultural Research Service, National Center for Agricultural Utilization Research, 1815 N. University St., Peoria, IL 61604, USA; Email: michael.jackson@ars.usda.gov

**Keywords:** ochratoxin A, nanosponge, isotherm analysis, red wine, decontamination

## Abstract

The ability of a cyclodextrin-polyurethane polymer to remove ochratoxin A from aqueous solutions was examined by batch rebinding assays. The results from the aqueous binding studies were fit to two parameter models to gain insight into the interaction of ochratoxin A with the nanosponge material. The ochratoxin A sorption data fit well to the heterogeneous Freundlich isotherm model. The polymer was less effective at binding ochratoxin A in high pH buffer (9.5) under conditions where ochratoxin A exists predominantly in the dianionic state. Batch rebinding assays in red wine indicate the polymer is able to remove significant levels of ochratoxin A from spiked solutions between 1–10 μg·L^−1^. These results suggest cyclodextrin nanosponge materials are suitable to reduce levels of ochratoxin A from spiked aqueous solutions and red wine samples.

## 1. Introduction

**Figure 1:**
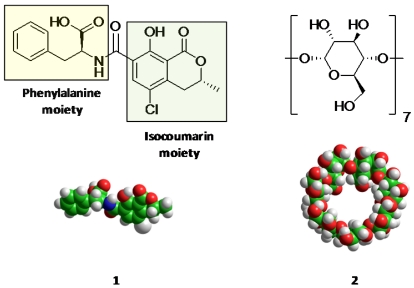
Representation of Ochratoxin A (OTA) (**1**) and β-cyclodextrin (**2**).

Ochratoxin A (OTA) is a secondary metabolite produced by certain fungi of the *Aspergillus *and *Penicillium* species which frequently contaminate a diverse range of agricultural commodities, including fruits, wines, coffee beans, and cereal grains [1,2,3]. Exposure to this toxin (see Figure 1) is associated with several deleterious effects on consumers, including, nephrotoxicity, neurotoxicity, teratogenicity, immunosuppression, and carcinogenicity [4]. The need to reduce exposure to mycotoxins has driven the investigation of several biological approaches and inert materials to reduce levels in commodities [5,6]. Some materials to selectively bind OTA and reduce free levels in solution include activated charcoal, grape pomace, and novel carbohydrates such as β-D-glucans isolated from *Saccharomyces cerevisiae *[7,8,9]. Recent interest in materials capable of selective interaction with OTA through molecular recognition mechanisms has driven the development of molecular imprinted polymers and aptamers. These selective recognition materials have enabled more robust analytical detection methods, and this molecular recognition approach has promise for other useful applications to reduce exposure to OTA [10,11,12]. 

Cyclodextrin nanosponge materials are a rapidly developing class of novel sorbents capable of molecular recognition [13,14,15,16]. These polymers include cyclodextrin components that possess hydrophobic binding site cavities of an appropriate size to form inclusion complexes with certain moieties of small organic molecules, including ochratoxins. β-cyclodextrin, **2**, is the most economical and commonly used cyclodextrin and features seven α-glucopyranose residues in the cyclic carbohydrate structure. The nanosponge materials have seen popular use as binders with “generic” binding sites able to remove a broad range of contaminants from aqueous solutions. The binding properties of these sites are susceptible to modulation by solvent and other factors. The cyclodextrin nanosponge materials are characterized by possessing very low surface areas by nitrogen adsorption BET (Brunauer-Emmett-Teller) surface area analysis and exceptional capacity for certain small organic molecules [13,15,16,17]. 

Free cyclodextrins have assisted in the recognition and detection of ochratoxins in analytical methods, and the ochratoxin-cyclodextrin guest-host complex has been characterized using spectroscopic and *in silico* molecular modeling techniques [18,19,20,21,22,23]. Spectrofluorimetric methods indicate OTA forms a 1:1 complex with β-cyclodextrin, and the interaction is influenced by the anionic and dianionic states of the toxin [21,23]. The interactions between OTA and cyclodextrins have been characterized using HINT natural force field calculations and circular dichroism experiments [18]. Furthermore, β-cyclodextrin has been used as a mobile phase component to assist the selective detection of zearalenone and OTA in HPLC methods [22] and to detect moniliformin, zearalenone, and ochratoxin A and B in a capillary electrophoresis method [19].

In this report, we expand the application of cyclodextrin-OTA complexes by using β-cyclodextrin as a critical component in a highly crosslinked polyurethane polymer sorbent. This insoluble polymer is evaluated for the ability to reduce levels of OTA in aqueous solutions. The OTA binding results from batch rebinding assays are fitted according to Langmuir and Freundlich two parameter-binding models. The polymer is demonstrated to reduce significant levels of OTA in red table wine. 

## 2. Materials and Methods

### 2.1. Chemicals

β-Cyclodextrin, tolylene 2,4-diisocyanate, acetic acid, anhydrous dimethyl formamide, activated charcoal, white quartz sand, silica, phosphoric acid, monobasic sodium phosphate, dibasic sodium phosphate, sodium hydroxide and OTA (from *Petromyces albertensis*, >98% TLC) were purchased from Sigma-Aldrich (St. Louis, USA). Acetonitrile, acetone, ethanol and methanol were purchased from EMB (Gibbstown, USA). Deionized water was used in the preparation of all reagents (Nanopure II, Sybron/Barnstead). All solvents were HPLC grade. The standard stock solution was prepared by dissolving 1 mg of OTA in 1 mL of methanol. 

### 2.2. Polymer Synthesis

Polymers were synthesized following modifications to published procedures [13,15]. In a 40 mL glass vial, β-cyclodextrin (2.0 mmol, 2.27 g) was dissolved in anhydrous dimethyl formamide (25 mL) by sonication for 15 min. The solution was flushed with a stream of nitrogen in a fume hood and 2.88 mL of tolylene 2,4-diisocyanate was added. The vial was sealed with a screw top cap, and the mixture was vortexed. The solution was sonicated for 15 min, and placed in a water bath at 70 °C. After 48 h, the excess reagents were removed from the monolith-gel by vacuum filtration and washing with excess acetone. The monoliths were sonicated in water, ethanol, and acetonitrile, then filtered, and dried under vacuum. Finally, the polymer was ground in a coffee grinder, sieved, and the 38–75 µm fractions were collected. Fine particulates were removed by sedimentation in acetone for 30 min (3 × 100 mL). The final particles were filtered, washed with excess water, and dried under vacuum.

### 2.3. Surface Analysis and Scanning Electron Microscopy

Surface analysis of the sorbent was performed on a Quantachrome Instruments ASiQ surface area analyzer. The sample (about 1 g) was outgassed at 100 °C for 24 h prior to analysis. The measurement was made using N_2_ as the adsorptive and the sample at −196 °C. Surface area was determined using the multipoint BET method in the relative pressure (*P*/*P*_o_) range of 0.05–0.20. SEM analysis was carried out using a Jeol JSM 6400V scanning electron microscope. 

### 2.4. Batch Rebinding Assays

The β-cyclodextrin-polyurethane polymer was evaluated in batch rebinding assays for the ability to remove OTA from aqueous solutions and red wine samples. For the experiments carried out in water and buffer, insoluble polymer (2 mg unless otherwise noted) was incubated in 1 mL solutions of OTA in water (0.01–5 μg·mL^−1^) or buffer (0.01–1 μg·mL^−1^). Samples were shaken on a Lab-line Multi-wrist shaker at room temperature for 24 h. The vials were centrifuged and supernatant was filtered through 0.2 μm filters (PTFE). All experiments were performed in triplicate. OTA concentrations in water were determined using peak areas and standard curves determined in water within the range of 0.0001–5 μg·mL^−1^ and buffer within a range of 0.0005–1 μg·mL^−1^ (*r*^2^*_water_* = 0.998; *r*^2^_pH 3.5_ = 0.999; *r*^2^_pH 7.0_ = 0.973; *r*^2^_pH 9.5_ = 0.995). The bound OTA was calculated by subtracting the amount of OTA free in solution at equilibrium in presence of the polymer from the initial OTA concentration. 

The batching rebinding assay results were analyzed using the Langmuir and Freundlich isotherms. The Langmuir equation is expressed as:

*q*_e_ = (*Q*_0_*K*_L_*C*_e_)/(1 + *K*_L_*C*_e_)

where *q*_e_ is the amount of OTA bound (mg) per polymer (g) at equilibrium. *C*_e_ is the amount of free OTA (mg) in solution at equilibrium. Q_0_ is the calculated maximum amount of OTA bound per gram of polymer, and *K*_L_ is the Langmuir equilibrium constant (L·mg^−1^) [24]. 

The Freundlich equation is described as:

*q*_e_ = *K*_f_*C*_e_^1/*n*^

*K*_f_ is the Freundlich constant, which is attributed to affinity and the adsorptive capacity of the polymer. The heterogeneity index, *n*, provides information on the population of the binding sites, the adsorption intensity, and is associated with the favorability of the binding process. A value of *n *= 1 suggests the population of binding sites is homogeneous [25]. 

### 2.5. LC-Analysis

OTA levels in aqueous solutions and red wine samples were determined by LC-fluorescence analysis. The HPLC consisted of a Shimadzu LC-20AT pump, a Rheodyne 7725 manual injector with a 20 μL injection loop, a RA-10 fluorescence detector, a CBM-20A communication bus model, and a Phenomenex Luna 5 μm C18 (2) 100A column (250 × 4.6 mm). The LC-mobile phase consisted of acetonitrile/water/acetic acid (49.5:49.5:1). The flow rate was 1 mL·min^−1^ and the fluorescence detector was set with the excitation wavelength of 333 nm and emission recorded at 460 nm. OTA eluted at 14 min. 

### 2.6. Ochratoxin A Determination in Red Wine

OTA concentrations in spiked red wine were determined with the following modifications to a published validated procedure [26]. Red table wine was purchased locally. OTA was isolated using C18 Bond Elut (500 mg) columns and a vacuum manifold prior to analysis. Columns were conditioned with 5 mL of methanol and 5 mL of water prior to extraction. Wine sample (1 mL) was filtered through a 0.2 µm syringe filter (PTFE) and combined with (5 mL) water, and passed through the C18 columns. The column was washed with 2 mL water, and 2 mL (60/40 methanol/water), and dried under vacuum. OTA was eluted with methanol (2 mL), and the eluate was filtered through a 0.2 μm syringe filter (PTFE) prior to analysis. OTA concentrations in wine were determined using peak areas and standard curves determined in wine within the range of 0.0005–0.010 μg·mL^−1^ for wine (*r*^2^ = 0.997). The bound OTA was calculated by subtracting the amount of OTA detected free in polymer solutions from the amount of OTA detected in wine standard solutions without polymer. 

## 3. Results and Discussion

**Figure 2:**
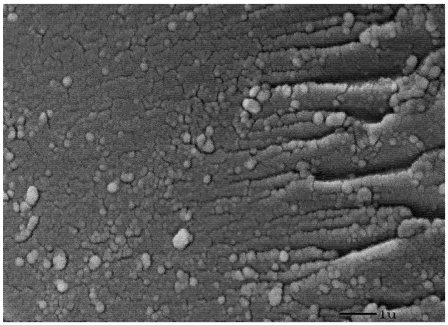
SEM image of a cross section of the β-cyclodextrin-polyurethane polymer at magnification of 10,000 times.

Crosslinked nanosponge polymers similar to the β-cyclodextrin-polyurethane polymer reported have exhibited the ability to remove significant levels of phenols, parabens, and other organic compounds from aqueous solutions and wastewater [27,28]. These cyclodextrin-polyurethane polymers have been characterized by FTIR, BET nitrogen surface area, and elemental analysis [13,17], and related cyclodextrin polymers have been extensively characterized [29]. The surface features of the polymer in this study are provided in the SEM image in Figure 2. The polymer has a smooth surface with channels of various sizes. 

**Figure 3:**
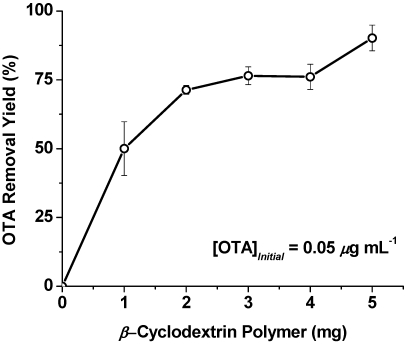
Effect of β-cyclodextrin-polyurethane polymer amount on OTA sorption in water (1 mL). The values are mean ± standard deviation.

The binding properties of the β-cyclodextrin-polyurethane polymer in this study were investigated using sorption isotherm analysis to gain insight into the OTA binding mechanism. The experiments reported here were carried out with a 24 h incubation period. We did investigate the influence of time on the sorption properties, and found that shorter incubation times did not provide maximum adsorption of OTA (15 min to 8 h). Reproducible binding studies were obtained with 24 h incubation. Figure 3 shows the influence of polymer mass on the sorption of OTA (0.05 μg·mL^−1^) carried out with 1 mL deionized water samples. A significant amount of OTA is sorbed at a polymer level of 2 mg, and the rest of the experiments described in this paper are carried out at this level. The level of 2 mg·mL^−1^ is significantly lower than levels of similar polymers used in related sorption assays. The levels used in this study are fivefold lower compared to the levels in a recent patulin study [13] and significantly less than the polymer levels to remove phenolic compounds from wastewater [28]. 

**Figure 4:**
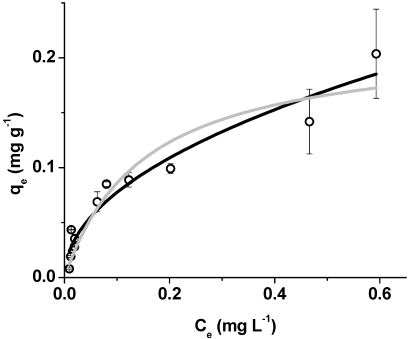
Freundlich (black) and Langmuir (silver) isotherms for OTA binding to the β-cyclodextrin-polyurethane polymer in water. The values are mean ± standard deviation.

The sorption isotherms for OTA binding to the polymer in water for the range of 0.01–1 μg·mL^−1^ are provided in Figure 4. These OTA concentrations are above the recommended levels for agricultural commodities (2–10 μg kg^−1^) fixed by the European Commission [21]. It should be noted, the polymer exhibited near complete removal at low levels of OTA. OTA levels lower than the recommended levels (2–10 μg L^−1^) are at levels so low that the bound OTA, *q*_e_, is very close to the origin in the plot and these levels do not contribute significantly to the Freundlich and Langmuir isotherms of the data of the range in Figure 4. The batch rebinding results in the range of 0.01–1 μg·mL^−1^ are suitable for fit to the Langmuir and Freundlich isotherms. Generally, the Langmuir model has several restrictions, and the Freundlich isotherm is more suitable for heterogeneous populations of binding sites. The Langmuir model is constrained by the several assumptions, including sorption is restricted to a monolayer of adsorbate, the binding sites are equivalent, and there is no cooperativity in binding [24]. The assumptions of the Langmuir model fit a dynamic binding process where the initial rate of binding is high, and the rate of sorption is related to the fraction of binding sites occupied. As binding sites become occupied, the rate of sorption is reduced as the number of free binding sites is decreased. Langmuir isotherm analysis of the binding results provides the Langmuir affinity constant of 6.60 L·mg^−1^ and the maximum OTA bound per gram of polymer is calculated to be 0.22 mg g^−1^ (see Table 1). 

**Table 1:** Isotherm parameters obtained by fitting binding data with the Langmuir and Freundlich isotherms for the sorption of ochratoxin A on β-cyclodextrin-polyurethane polymer in deionized water.

Model			*R*^2^
Langmuir	*K*_L_ (L·mg^−1^)	*Q*_0_ (mg·g^−1^)	
	6.60 ± 2.24	0.22 ± 0.03	0.909
Freundlich	*K*_F_ (mg·g^−1^)(L·mg^−1^)^1/*n*^	1/*n*	
	0.24 ± 0.02	0.49 ± 0.05	0.946

The Freundlich isotherm describes non-ideal sorption on heterogeneous surfaces and is capable of describing multi-layer and other moderately complicated binding processes [24]. The value of 0.49 for 1/*n* is associated with a mild rise of the sorption isotherm and favorable binding over the range of concentrations studied [25]. Comparing the results from the binding studies, the Freundlich analysis provides a tighter fit than the Langmuir model based on the determination coefficients (*R*^2^). This may be explained by the binding process occurring under conditions outside of the assumptions of the Langmuir model. This is supported with the heterogeneity index, which suggests the β-cyclodextrin-polyurethane polymer possesses a population of binding sites for OTA, a property more fit for Freundlich analysis.

**Figure 5:**
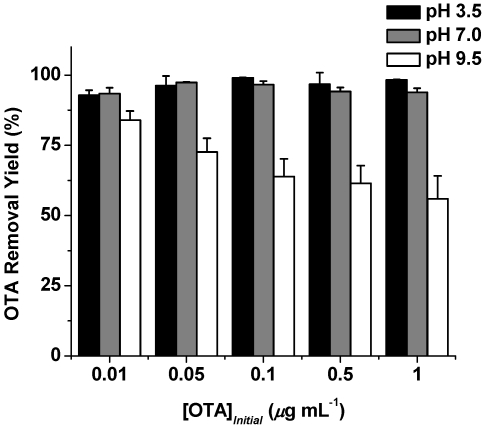
Influence of pH on the OTA removal yield (%) in the presence of β-cyclodextrin-polyurethane polymer (2 mg·mL^−1^) in sodium phosphate buffer (20 mM) over concentrations of OTA. ([OTA]_intial_ = 0.01–1 μg·mL^−1^). The values are mean ± standard deviation.

The sorptive capacity of broad classes of sorbents, including cyclodextrin polymers, can be influenced pH and other factors [30]. The adsorptive capacity of cyclodextrin-based materials for the basic dye C.I. Basic Green 4 (Malachite Green) has been observed to be pH dependent [31]. Solid phase extraction recoveries of nitrophenols in water by β-cyclodextrin bonded silica have been shown to be influenced by pH [32]. OTA possesses acidic phenolic and carboxylic acid groups, and the nature of OTA is dependent on pH. To gain insight into the influence of pH on OTA binding to the β-cyclodextrin-polyurethane polymer, batch rebinding assays were carried out over OTA concentrations of 0.01–1 μg·mL^−1^ and pH values of 3.5, 7.0, and 9.5 (see Figure 5). The β-cyclodextrin-polyurethane polymer (2 mg·mL^−1^) binds almost all OTA at the concentrations between 0.01–1 μg·mL^−1^ at pH values 3.5 and 7.0. There is a decrease in capacity of the polymer for OTA at the higher pH 9.5 where OTA predominantly exists in the dianionic form, suggesting a lesser amount of the dianionic OTA is bound to the polymer. The lack of activity may be attributed to the repulsive interactions of the bound to the free OTA in the dianionic state. In contrast, the two negatively charged groups are not present in the neutral free acid OTA and the anionic OTA.

It should be noted OTA possesses a greater affinity to free β-cyclodextrin at higher pH [18,21,23], which has been rationalized by the favorable interaction of the hydroxyls of the free β-cyclodextrin with the dianionic form of OTA. The hydroxyls of the cyclodextrin components of the β-cyclodextrin-polyurethane polymer are reacted to form polyurethane residues, and the reduced number of these hydroxyls in the polymer may explain the decrease in OTA binding at higher pH levels. The increased capacity of the β-cyclodextrin-polyurethane polymer for OTA under acidic is a valuable property to reduce OTA levels in acidic beverages, such as juices and wine. 

The efficiency of the β-cyclodextrin-polyurethane polymer to remove OTA from red table wine is shown in Table 2. Spiked wine levels between 1–10 μg L^−1 ^were investigated after 24 h incubation with the polymer. The β-cyclodextrin-polyurethane polymer (2 mg·mL^−1^) is capable of removing significant amount of OTA from spiked wine samples. The best activity of the polymer was between 2.5–10 μg·L^−1^. The lower percent bound at 1 μg·L^−1^ (61%) may be associated with the OTA levels approaching the level of detection of the analytical method. In addition, there may be competition for the binding sites of the polymer by other wine constituents of similar molecular size at low levels of OTA, a phenomenon that is expected to increase in importance as the relative levels of competing substrates for the binding sites increases at lower levels of OTA. 

**Table 2:** Sorption of ochratoxin A in red wine by β-cyclodextrin-polyurethane polymer ^a^.

[OTA] *_Initial_* (μg L^−1^)	OTA*_Bound_*^b^ (μg)	% OTA*_Bound_*
10	8.82 ± 0.23	88
7.5	7.11 ± 0.11	95
5	4.62 ± 0.20	92
2.5	2.33 ± 0.12	93
1	± 0.14	61

^a^ Red wine samples were spiked with OTA and shaken for 24 h prior to analysis; ^b^ The values are mean ± standard deviation.

The binding activity exhibited by the β-cyclodextrin-polyurethane polymer approaches the sorption activity of activated charcoal (9.42 ± 0.09 for [OTA]*_Initial_* = 10 μg L^−1^; 2 mg·mL^−1^ activated charcoal). However, activated charcoal possesses an average surface area over 1090 m^2^·g^−1^ as determined by nitrogen adsorption BET analysis [33]. Other materials we investigated with various surfaces areas, sand and 200–400 mesh silica, did not reduce detectable levels of OTA in red wine (10 μg·L^−1^) in the 24 h batch rebinding assays using 2 mg·mL^−1^ of material. The surface area of the polymer evaluated in this study was calculated to be 0.759 m^2^·g^−1^ and is similar to previous published surface areas for related nanosponge materials [13,17]. This β-cyclodextrin-polyurethane polymer provides a low surface area alternative to activated charcoal with suitable OTA binding activity for levels below 10 μg·L^−1^ in red wine. 

The non-selective binding of valuable components of beverages by sorbents is an important consideration. The “generic binding sites” of the β-cyclodextrin-polyurethane are expected to be susceptible to binding and removal a broad range of substrates with OTA. It remains to be shown if nanosponge materials can be optimized using select components for more selective removal of certain toxins over other constituents from aqueous solutions. One significant advantage of the β-cyclodextrin-polyurethane polymer over the use of free cyclodextrins is the β-cyclodextrin-polyurethane polymer is insoluble in aqueous solutions, permitting rapid separation of the material and toxin from aqueous solutions. Furthermore, the polymer is synthesized as a monolithic block, allowing for the possible inclusion of other sorbent components, such as powder charcoals. In addition, the β-cyclodextrin-polyurethane particles can be ground to a desired size (38–75 µm in this study) or synthesized as films to support easy separation of the sorbent from solutions. 

## 4. Conclusions

A nanosponge polymer composed of β-cyclodextrin-polyurethane was evaluated for the ability to remove levels of OTA from aqueous solutions and red wine. Analysis of the Langmuir isotherm of the OTA binding studies in water indicates the polymer has a maximum capacity of 0.22 mg OTA per gram of polymer. Results of the Freundlich isotherm suggest the polymer possesses a heterogeneous population of binding sites for OTA. The polymer was capable of reducing levels of OTA up to 10 μg·L^−1^ in spiked red wine samples to levels below recommended levels (2 μg L^−1^). This study suggests nanosponge materials can assist reducing levels of natural product contaminants, including OTA, in beverages. 

## References

[1] Duarte S.C., Lino C.M., Pena A. (2010). Mycotoxin food and feed regulation and the specific case of ochratoxin A: A review of the worldwide status.. Food Addit. Contam..

[2] El Khoury A., Atoui A. (2010). Ochratoxin A: General overview and actual molecular status.. Toxins.

[3] Tozlovanu M., Pfohl-Leszkowicz A. (2010). Ochratoxin A in roasted coffee from french supermarkets and transfer in coffee beverages: Comparison of analysis methods.. Toxins.

[4] Varga J., Kocsubé S., Péteri Z., Vágvölgyi C., Tóth B. (2010). Chemical, physical and biological approaches to prevent ochratoxin induced toxicoses in humans and animals. Toxins.

[5] Abrunhosa L., Paterson R., Venâncio A. (2010). Biodegradation of ochratoxin A for food and feed decontamination. Toxins.

[6] Amézqueta S., González-Peñas E., Murillo-Arbizu M., López de Cerain A. (2009). Ochratoxin A decontamination: A review. Food Control.

[7] Espejo F., Armada S. (2009). Effect of activated carbon on ochratoxin A reduction in “Pedro Ximenez” sweet wine made from off-vine dried grapes.. Eur. Food Res. Technol..

[8] Solfrizzo M., Avantaggiato G., Panzarini G., Visconti A. (2009). Removal of ochratoxin A from contaminated red wines by repassage over grape pomaces.. J. Agric. Food Chem..

[9] Yiannikouris A., André G., Poughon L., François J., Dussap C.-G., Jeminet G., Bertin G., Jouany J.-P. (2006). Chemical and conformational study of the interactions involved in mycotoxin complexation with β-D-glucans.. Biomacromolecules.

[10] Bazin I., Nabais E., Lopez-Ferber M. (2010). Rapid visual tests: Fast and reliable detection of ochratoxin A.. Toxins.

[11] De Girolamo A., McKeague M., Miller J.D., DeRosa M.C., Visconti A. (2011). Determination of ochratoxin A in wheat after clean-up through a DNA aptamer-based solid phase extraction column.. Food Chem..

[12] Yu J.C.C., Lai E.P.C. (2010). Molecularly imprinted polymers for ochratoxin A extraction and analysis.. Toxins.

[13] Appell M., Jackson M. (2010). Synthesis and evaluation of cyclodextrin-based polymers for patulin extraction from aqueous solutions. J. Incl. Phenom. Macrocycl. Chem..

[14] Mele A., Castiglione F., Malpezzi L., Ganazzoli F., Raffaini G., Trotta F., Rossi B., Fontana A., Giunchi G. (2011). HR MAS NMR, powder XRD and Raman spectroscopy study of inclusion phenomena in βCD nanosponges. J. Incl. Phenom. Macrocycl. Chem..

[15] Mhlanga S.D., Mamba B.B., Krause R.W., Malefetse T.J. (2007). Removal of organic contaminants from water using nanosponge cyclodextrin polyurethanes.. J. Chem. Technol. Biotechnol..

[16] Xiao P., Dudal Y., Corvini P.F.X., Pieles U., Shahgaldian P. (2011). Cyclodextrin-based polyurethanes act as selective molecular recognition materials of active pharmaceutical ingredients (APIs). Polym. Chem..

[17] Wilson L.D., Mohamed M.H., Headley J.V. (2011). Surface area and pore structure properties of urethane-based copolymers containing [beta]-cyclodextrin.. J. Colloid Interface Sci..

[18] Amadasi A., Dall’Asta C., Ingletto G., Pela R., Marchelli R., Cozzini P. (2007). Explaining cyclodextrin-mycotoxin interactions using a “natural” force field.. Bioorg. Med. Chem..

[19] Böhs B., Seidel V., Lindner W. (1995). Analysis of selected mycotoxins by capillary electrophoresis.. Chromatographia.

[20] Cozzini P., Ingletto G., Singh R., Dall’Asta C. (2008). Mycotoxin detection plays “cops and robbers”: Cyclodextrin chemosensors as specialized police?. Int. J. Mol. Sci..

[21] Hashemi J., Alizadeh N. (2009). Investigation of solvent effect and cyclodextrins on fluorescence properties of ochratoxin A.. Spectrochim. Acta Part A.

[22] Seidel V., Poglits E., Schiller K., Lindner W. (1993). Simultaneous determination of ochratoxin A and zearalenone in maize by reversed-phase high-performance liquid chromatography with fluorescence detectio.. J. Chromatogr. A.

[23] Verrone R., Catucci L., Cosma P., Fini P., Agostiano A., Lippolis V., Pascale M. (2007). Effect of β-cyclodextrin on spectroscopic properties of ochratoxin A in aqueous solution.. J. Incl. Phenom. Macrocycl. Chem..

[24] Foo K.Y., Hameed B.H. (2010). Insights into the modeling of adsorption isotherm systems.. Chem. Eng. J..

[25] Tseng R.-L., Wu F.-C. (2008). Inferring the favorable adsorption level and the concurrent multi-stage process with the Freundlich constant.. J. Hazard. Mater..

[26] Hernández M.J., García-Moreno M.V., Durán E., Guillén D., Barroso C.G. (2006). Validation of two analytical methods for the determination of ochratoxin A by reversed-phased high-performance liquid chromatography coupled to fluorescence detection in musts and sweet wines from Andalusia.. Anal. Chim. Acta.

[27] Chin Y.P., Mohamad S., Abas M.R.B. (2010). Removal of parabens from aqueous solution using β-cyclodextrin cross-linked polymer. Int. J. Mol. Sci..

[28] Yamasaki H., Makihata Y., Fukunaga K. (2006). Efficient phenol removal of wastewater from phenolic resin plants using crosslinked cyclodextrin particles.. J. Chem. Technol. Biotechnol..

[29] Salipira K.L., Krause R.W., Mamba B.B., Malefetse T.J., Cele L.M., Durbach S.H. (2008). Cyclodextrin polyurethanes polymerized with multi-walled carbon nanotubes: Synthesis and characterization.. Mater. Chem. Phys..

[30] Crini G. (2005). Recent developments in polysaccharide-based materials used as adsorbents in wastewater treatment.. Prog. Polym. Sci..

[31] Crini G., Peindy H.N., Gimbert F., Robert C. (2007). Removal of C.I. Basic Green 4 (Malachite Green) from aqueous solutions by adsorption using cyclodextrin-based adsorbent: Kinetic and equilibrium studies. Sep. Purif. Technol..

[32] Fan Y., Feng Y.-Q., Da S.-L., Feng P.-Y. (2003). Evaluation of beta-cyclodextrin bonded silica as a selective sorbent for the solid-phase extraction of 4-nitrophenol and 2,4-dinitrophenol.. Anal. Sci..

[33] Putra E.K., Pranowo R., Sunarso J., Indraswati N., Ismadji S. (2009). Performance of activated carbon and bentonite for adsorption of amoxicillin from wastewater: Mechanisms, isotherms and kinetics. Water Res..

